# A Mechanistic Model of Aqueous Humor Flow to Study Effects of Angle Closure on Intraocular Pressure

**DOI:** 10.1167/tvst.12.1.16

**Published:** 2023-01-09

**Authors:** Javier Murgoitio-Esandi, Benjamin Y. Xu, Brian J. Song, Qifa Zhou, Assad A. Oberai

**Affiliations:** 1 Department of Aerospace and Mechanical Engineering, Viterbi School of Engineering, University of Southern California, Los Angeles, CA, USA; 2 USC Roski Eye Institute, Department of Ophthalmology, Keck School of Medicine, University of Southern California, Los Angeles CA, USA; 3 Department of Biomedical Engineering, Viterbi School of Engineering, University of Southern California, Los Angeles CA, USA

**Keywords:** ocular fluid dynamics, angle closure, intraocular pressure, aqueous humor flow

## Abstract

**Purpose:**

To study the relationship between the circumferential extent of angle closure and elevation in intraocular pressure (IOP) using a novel mechanistic model of aqueous humor (AH) flow.

**Methods:**

AH flow through conventional and unconventional outflow pathways was modeled using the unified Stokes and Darcy equations, which were solved using the finite element method. The severity and circumferential extent of angle closure were modeled by lowering the permeability of the outflow pathways. The IOP predicted by the model was compared with biometric and IOP data from the Chinese American Eye Study, wherein the circumferential extent of angle closure was determined using anterior segment OCT measurements of angle opening distance.

**Results:**

The mechanistic model predicted an initial linear rise in IOP with increasing extent of angle closure which became nonlinear when the extent of closure exceeded around one-half of the circumference. The nonlinear rise in IOP was associated with a nonlinear increase in AH outflow velocity in the open regions of the angle. These predictions were consistent with the nonlinear relationship between angle closure and IOP observed in the clinical data.

**Conclusions:**

IOP increases rapidly when the circumferential extent of angle closure exceeds 180°. Residual AH outflow may explain why not all angle closure eyes develop elevated IOP when angle closure is extensive.

**Translational Relevance:**

This study provides insight into the extent of angle closure that is clinically relevant and confers increased risk of elevated IOP. The proposed model can be utilized to study other mechanisms of impaired aqueous outflow.

## Introduction

Glaucoma is the most common cause of permanent vision loss worldwide.^1^ Elevated intraocular pressure (IOP) is an important risk factor for glaucomatous optic neuropathy and contributes to irreversible damage of retinal ganglion cell axons.^2^ Primary glaucoma is divided into two broad categories: primary open-angle glaucoma (POAG) and primary angle-closure glaucoma (PACG). PACG is characterized by narrowing and closure of the anterior chamber angle, the primary site of aqueous outflow from the eye. PACG carries a threefold increased risk for severe bilateral visual impairment compared to POAG and is associated with severe ocular morbidity and high rates of unilateral blindness on diagnosis.^3^^–^^6^

**Figure 1.**
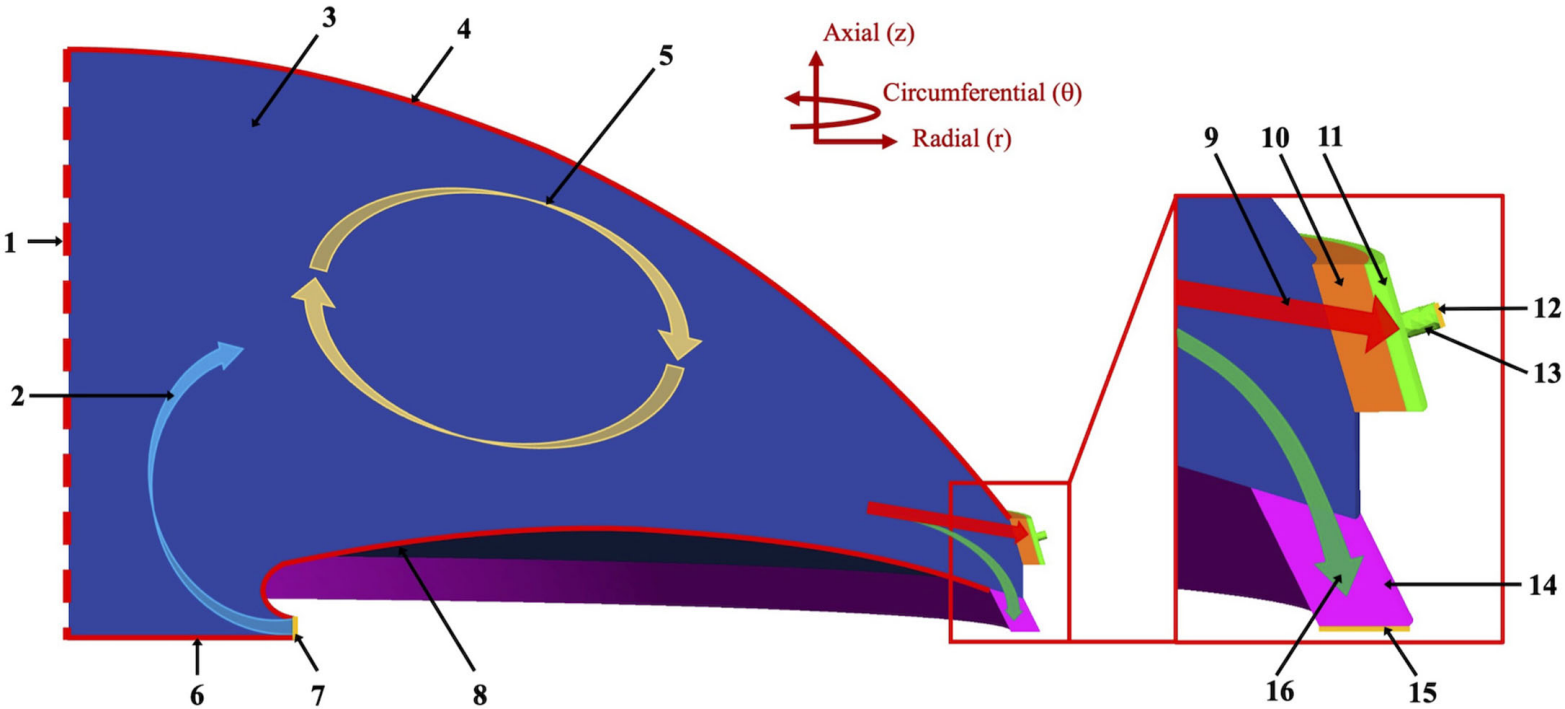
Illustrative section of the flow throughout the computational domain. (1) Eye centerline; (2) aqueous humor inflow into the model; (3) anterior chamber; (4) zero-flow boundary with the cornea; (5) buoyancy-driven vortex; (6) zero-flow boundary with the lens; (7) aqueous humor inflow surface; (8) zero-flow boundary with the iris; (9) conventional outflow pathway; (10) trabecular meshwork; (11) Schlemm's canal; (12) conventional outflow boundary; (13) collector channel; (14) ciliary body; (15) unconventional outflow boundary; (16) unconventional outflow pathway.

Aqueous humor (AH) is produced by the ciliary body (CB) epithelium and enters the posterior chamber at a rate of 1.4 to 4.3 µL/min.^7^ AH then flows between the lens and the iris, around the pupillary margin, and into the anterior chamber (AC). From the AC, AH exits the eye through either the trabecular meshwork (TM; conventional outflow) or through the CB face (unconventional outflow) (Fig. 1). The unconventional outflow pathway comprises approximately 50% of outflow in young healthy individuals and declines with age at a rate of approximately 3.5% per decade,^8^ possibly due to a build-up of extracellular material, an increase in the number and thickness of collagen fibers, and a thickening of the elastic fibers, which results in reduced space between muscle bundles.^9^ Angle closure, characterized by apposition between the peripheral iris and TM, leads to mechanical obstruction of both outflow pathways. This contributes to increased outflow resistance and increase in IOP, as AH production is mostly independent of IOP.

In this study, we developed a novel mechanistic model of AH flow that can model impaired outflow through both conventional and unconventional outflow pathways. We used the model to assess the relationship between angle closure and IOP. We then compared and validated the model predictions with data from the Chinese American Eye Study (CHES), a population-based epidemiologic study on ocular disease. Although prior models have been developed to understand the pathogenesis of elevated IOP,^10^^–^^12^ and extensive work has been done in this area, there is relatively sparse information about the relation between the extent of angle closure and elevated IOP in humans.

## Methods

### Model Development

#### Geometry

**Figure 2.**
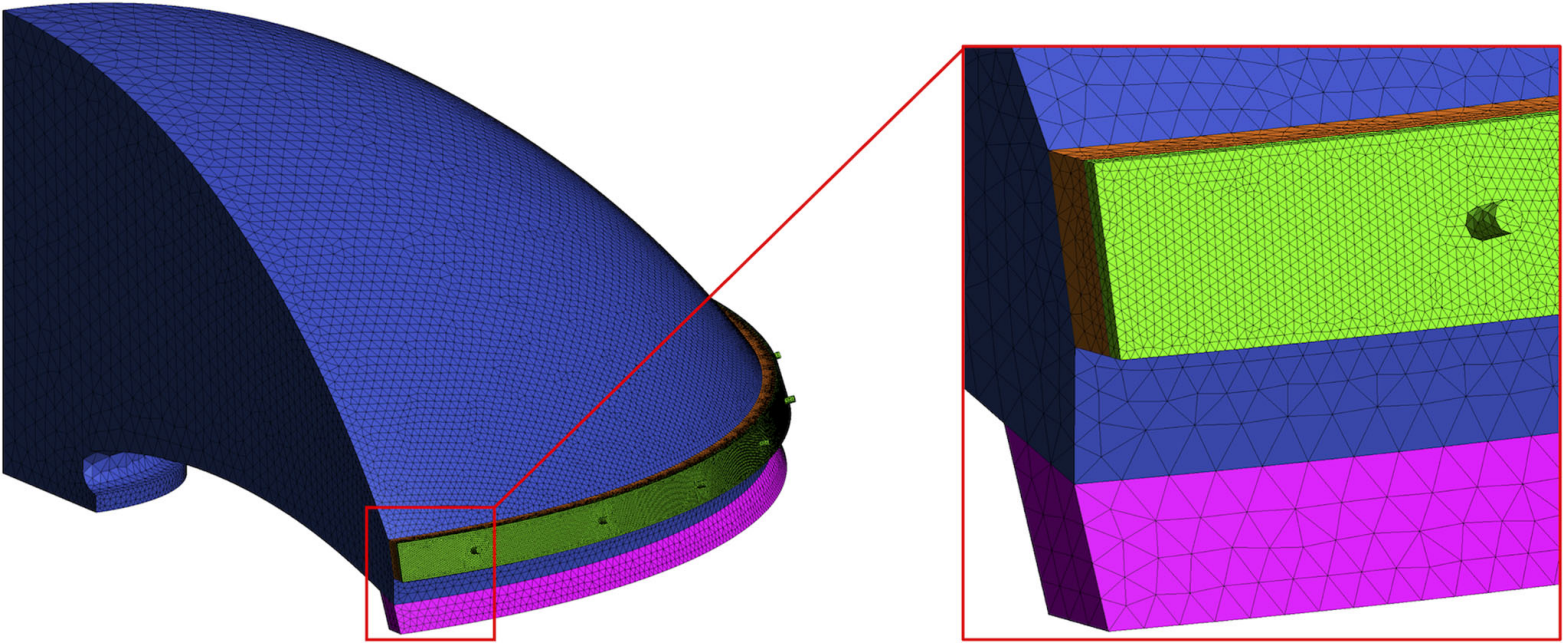
Image of the mesh used in the computations.

The model geometry consisted of a quarter of the AC in one eye of a person in the supine position. An overview of the geometry of the mechanistic model is represented in Figure 1. The geometric dimensions (AC height = 3.5 mm and AC diameter = 11.3 mm) were based on anterior chamber measurements,^13^ and the model was built using Gmsh 4.6.0. The entire model geometry is shown in Figure 2. Following Dautriche et al.,^14^ the TM was modeled as a uniform body with a rectangular cross-section of 275 µm (height in the axial direction, *z*) by 100 µm (length in the radial direction, *r*) and with uniformly distributed permeability. Therefore, the height of the TM was set equal to the height of the Schlemm's canal (SC), or 275 µm. The SC was also modeled as a uniform body with a rectangular cross-section of 275 µm by 30 µm, based on the work of Gong et al.^16^ Seven evenly distributed collector channels (CCs) with a diameter of 50 µm were placed along the outer wall of the SC.^16^ It was verified that the distribution and location of the CCs did not affect outflow or IOP (see Appendix A). The unconventional outflow path consisted primarily of the CB face, which allowed the AH to exit the eye through the AC angle. In the model, the CB face was modeled adjacent to the TM in the AC angle with uniformly distributed permeability. The outflow pathway to the vitreous body,^17^ which can be characterized as a porous domain,^18^ was not accounted for in the model, as that flow occurs prior to the AH inflow in our model. The finite element mesh contained 336,516 tetrahedral finite elements. Figure 2 shows an overview of the mesh.

#### Governing Equations

The flow of the AH was modeled using the unified Stokes and Darcy equations by considering the flow within the non-porous regions (i.e., AC, SC, and CCs) as Stokes flow regions and the flow within the porous regions (TM and CB) as Darcy flow. As described below, the permeability in the Darcy model was modeled to decrease with increasing IOP.^19^ Additionally, the convective and diffusive transport of energy due to the temperature difference between the cornea and internal surfaces was modeled by solving a convection–diffusion equation for the temperature. The model was developed in the finite-element solver FEniCS 2019.1.0.^20^ The model and the solver type chosen for this study allowed for an accurate representation of the AH outflow system and the ability to model angle closure.

##### Stokes Flow Region

The flow in this region is governed by incompressible steady Stokes equations (Equations 1 and 2), which are a simplification of the Navier–Stokes equation at low Reynolds number (Re):

(1) $$\begin{array}{c} -\mu \nabla^{2}u+\nabla p=f \end{array}$$

(2) $$\begin{array}{c} \nabla \cdot u=0, \end{array}$$

where µ is the fluid viscosity, ***u*** is the fluid velocity vector field, *p* is the scalar pressure field, and ***f*** is the body-force term, defined as

(3) $$\begin{array}{c} f=\rho g=\rho_{0}g\left[1-\gamma \left(T-T_{ref}\right)\right]. \end{array}$$

Here, ***g*** is the gravitational acceleration vector, ρ_0_ is the reference density, γ is the volume expansion coefficient, *T* is the temperature, and *T_ref_* is the reference temperature. It was verified that for this study the Re is sufficiently small (Re ≈ 0.15) and therefore the Stokes approximation is appropriate.

##### Flow Through the Porous Region (Darcy Model)

The flow through porous media is governed by the Darcy model:

(4) $$\begin{array}{c} u+\frac{K}{\mu }\nabla p=0 \end{array}$$

(5) $$\begin{array}{c} \nabla \cdot u=0, \end{array}$$

where *K* is the permeability of the medium. The Darcy model was modified to allow for decreasing permeability with increasing IOP:

(6) $$\begin{array}{c} \frac{K}{\mu }=\frac{\alpha }{1+\beta *p}, \end{array}$$

where µα is the permeability at zero pressure, and β determines the reduction in permeability with increasing IOP.^19^ In the TM, β is selected to simulate the mild decrease in permeability associated with increasing IOP (a drop of 19% as IOP increases from 15 to 30 mmHg). In the CB, β is selected to be sufficiently large so that $u\approx -\frac{\alpha }{\beta }\nabla p/p=-\frac{\alpha }{\beta }\nabla \ln\left(p\right)$, which is much weaker than the linear dependence of velocity on pressure gradient in other regions. This makes the unconventional outflow in our model weakly dependent on IOP, a phenomenon that is consistent with experimental studies.^21^^,^^22^

##### Unified Flow

The unified Stokes and Darcy equations (also called the Stokes–Brinkman model) are

(7) $$\begin{array}{c} -\mu \nabla^{2}u+Ku+\nabla p=\rho_{0}g\left[1-\gamma \left(T-T_{ref}\right)\right] \end{array}$$

(8) $$\begin{array}{c} \nabla \cdot u=0. \end{array}$$

In the porous regions, where the Darcy model is appropriate, the parameter µ is set to zero (***g*** is also assumed to be zero), and in the Stokes flow regions the parameter *K* is set to zero. Thus, this single model captures both the Stokes and Darcy flow models.

At the interface between the Stokes and Darcy flow regions, represented by the superscripts (1) and (2), respectively, we have ***u***^(1)^ = ***u***^(2)^. This is because we use continuous function spaces for the velocity field. Further, the Euler–Lagrange equations associated with the weak form of the Stokes–Brinkman equations yield:

(9) $$\begin{array}{c} p^{\left(1\right)}=p^{\left(2\right)} \end{array}$$

and

(10) $$\begin{array}{c} \left(2\mu^{\left(1\right)}\nabla^{s}u\right)*n\left(1\right)=0, \end{array}$$

where ∇^(^*^s^*^)^***u*** is the symmetric gradient of the velocity field, and ***n***^(1)^ is the normal vector pointed away from the Stokes region. Equation 9 indicates that the pressure is continuous across the interface, and Equation 10 reveals that at shear stress it is zero.

##### Convective and Diffusive Transport of Energy

The convective and diffusive transport of energy by the AH is described by the convection–diffusion equation below, which is solved together with the unified flow equations:

(11) $$\begin{array}{c} \rho C_{p}u\cdot \nabla T=k\nabla^{2}T \end{array}$$

where *C_p_* is the heat capacity, and *k* is the thermal conductivity.

#### Model Parameters

The parameters defining the AH flow were fixed in all simulations (Table 1). The permeability values were calibrated to match the modeled outflow facility with the outflow facility reported by Brubaker^23^ in enucleated human eyes (see Appendix B). The derived TM permeability parameter, α*_TM_*, was 4.5 × 10^−13^ m^2^, and the permeability parameter of the CB face was of the same order of magnitude. These values are in accordance with the findings of other numerical studies.^12^

**Table 1.** Model Parameters

Item	Magnitude
AH viscosity, µ	0.001 kg m^−1^ s^−1^
AH density, ρ	1000 kg m^−3^
AH thermal conductivity, *k*	0.6 W m^−1^ K^−1^
AH specific heat, *C_p_*	4182 J kg^−1^ K^−1^
Volume expansion coefficient, γ	0.0003 K^−1^
Episcleral venous pressure, *EVP*	933.254 Pa
α permeability parameter of TM, α*_TM_*	4.5 × 10^−13^ m^3^ s kg^−1^
α permeability parameter of CB, α*_CB_*	1.0 × 10^−12^ m^3^ s kg^−1^
β permeability parameter of TM, β*_TM_*	1.125 × 10^−4^ Pa^−1^
β permeability parameter of CB, β*_CB_*	3.75 × 10^−3^ Pa^−1^

#### Boundary Conditions

We assume that the flow of the AH into the iris, the cornea, and the lens is small compared to its flow through the conventional and unconventional pathways; therefore, we model these tissues with a zero-flow no-slip boundary condition. Flow velocity boundary conditions were divided among three areas, which consisted of the inflow boundary, located in between the iris and the lens; the outflow boundary, located at the CCs and the CB (see Fig. 1); and the no-slip boundary, which was comprised of all other boundaries. The inflow rate was set at 3 µL/min for all analyses. Outflow boundaries were defined by setting the fluid pressure at the outflow surface. The pressure at the conventional and unconventional outflow boundaries was set to 7 mmHg, representing the episcleral venous pressure.

The temperature boundary conditions were divided among the exterior boundary located at the cornea, which was set to 35°C, and the interior boundaries (everywhere except the cornea), which were set to 37°C. At the *x* = 0 mm and *y* = 0 mm planes, a symmetric boundary condition was applied, wherein the normal velocity and temperature gradient were set to zero.

#### Modeling Angle Closure

The mechanistic model was used to simulate angle closure by decreasing the permeability of the outflow pathways in the sectors with angle closure. The permeability was decreased by a factor (i.e., the outflow facility reduction factor) that was used as a surrogate for the severity of closure. As an example, we show the results of modeling an open-angle eye compared to modeling an eye with angle closure in one-half of its circumference. Here, the sectors with angle closure were modeled using an outflow facility reduction factor of 10; that is, the outflow facility was 10 times lower in the “closed” sectors.

**Figure 3.**
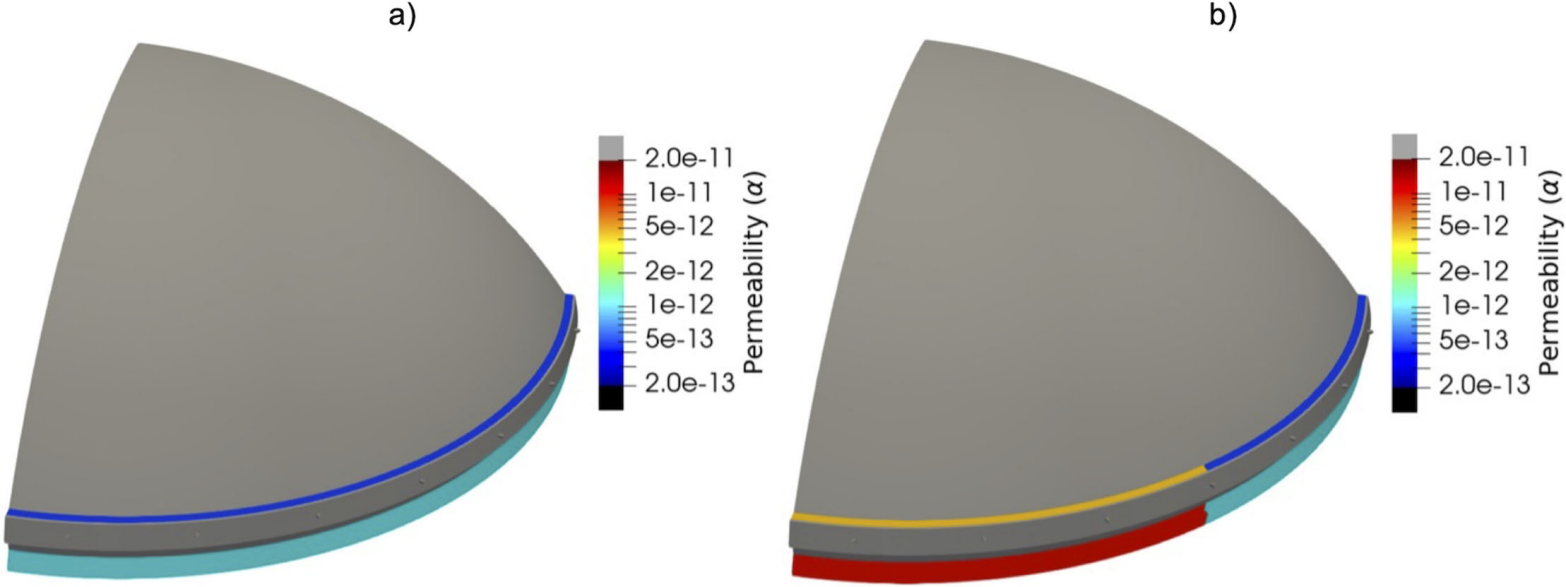
Distribution of permeability parameter α. (a) No angle closure present, and (b) angle closure present for half of the circumference.

**Figure 4.**
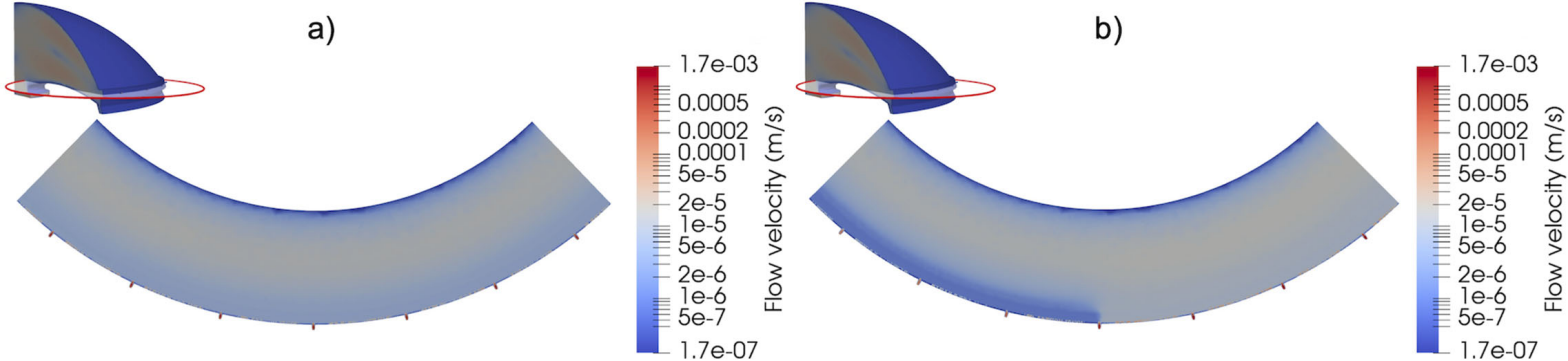
Flow velocity at a horizontal section of the anterior chamber near the TM and CCs. (a) No angle closure present, and (b) angle closure present along half of the circumference. The permeability in the “closed” sectors is 10 times smaller than in the healthy sectors.

Figure 3 shows the distribution of the permeability parameter α, which is the permeability parameter reduced by the outflow facility reduction factor. Figures 4a and 4b show the magnitude of the flow velocity in a cross-sectional view of the AC near the TM and CCs for the open-angle eye and the eye with angle closure, respectively. It can be observed that, although in the open-angle eye the flow is uniform through the circumference, in the eye with angle-closure the reduction of outflow facility impedes the AH flow through the “closed” sectors. As mentioned above, in the “closed” sectors the value of the permeability is reduced by a factor of 10 with respect to the healthy sectors. Also, the pressure drop across the outflow pathways for both healthy and unhealthy sectors is the same. Because the pressure drop is proportional to the ratio of the velocity and the permeability, in order to maintain the same pressure drop in the regions where the permeability is reduced by a factor of 10 the flow velocity is also reduced by a factor of 10. This is observed in Figure 4.

In addition, we note that angle closure was modeled by reducing permeability rather than completely closing the angle in the geometric model, because the full closure of the angle would completely block AH flow, which would imply that the iris is not permeable. However, it is known that the iris is permeable to AH,^24^ and even eyes with extensive gonioscopic iris–meshwork apposition sometimes have normal IOP levels,^25^ indicating the presence of residual AH flow. Thus, by reducing permeability we allow for residual AH flow, which can be regulated with the magnitude of the outflow facility reduction factor.

### Clinical Data

Clinical data were obtained from the CHES, a population-based epidemiological study of angle closure in Los Angeles, CA.^26^ Data from 2395 individual eyes from 2395 participants were used. Each CHES participant received a complete eye examination, including IOP checked using the Goldman Applanation Tonometer (Haag Streit USA, Mason, OH) and anterior segment OCT (AS-OCT) imaging using the CASIA SS-1000 (Tomey Corporation, Nagoya, Japan). Anterior segment parameters were measured in four AS-OCT images per eye.^27^ Table 2 describes the presence of peripheral anterior synechiae (PAS), primary angle closure suspect (PACS), and PACG in the eyes in the clinical data. Angle width in the form of angle opening distance at 500 µm from the scleral spur (AOD500) was measured in eight sectors evenly spaced 45° apart. The lateral resolution of the CASIA SS-1000 is about 30 µm; therefore, a threshold of 0.04 mm (slightly more than 1 pixel) was used to detect angle closure, as manual detection of iridotrabecular contact (ITC) was not performed. A secondary sensitivity analysis was performed with thresholds of 0.02 and 0.06 mm. Additional information on the clinical assessment and AS-OCT image analysis in CHES is provided in Xu et al.^28^

**Table 2.** PAS, PACS, and PACG Present in the Eyes in the Clinical Data

	PAS						
	0 Quarters	1 Quarter	2 Quarters	3 Quarters	4 Quarters	PACS	PACG
Number of eyes	2281	67	26	10	11	229	89

## Results

### Mechanistic Model

**Figure 5.**
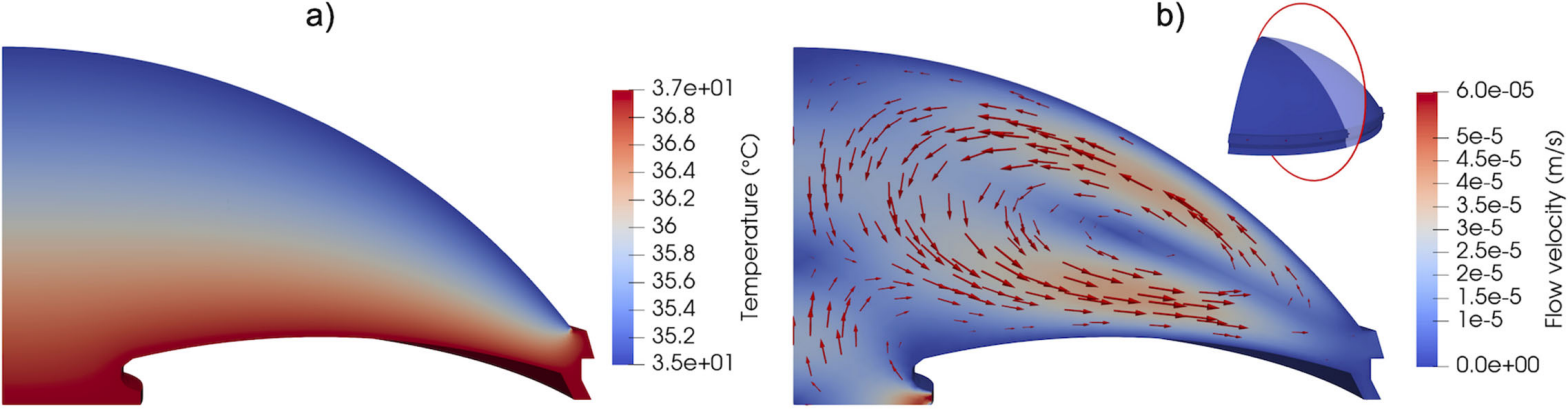
(a) Temperature distribution at the AC, and (b) section of flow velocity at the AC. The results were obtained using an inflow rate of 3.0 µL/min.

It has been experimentally observed that the flow of AH in the AC is dominated by a buoyancy-driven vortex,^29^ which is captured by our model. Figure 5a shows that the temperature distribution in the AC uniformly varies from 35°C at the outer boundary surface (cornea) to 37°C at the inner surfaces, and Figure 5b shows the flow velocity (both direction and magnitude) at a cross-section of the AC with an inflow rate of 0.75 µL/min (3 µL/min overall over all four quadrants). We note that the section of the flow shown in Figure 5b is representative of the radially symmetric flow in an eye with no angle closure. The buoyancy-driven vortex shown in Figure 5b is comprised of a flow moving downward at the center of the AC, outward near the iris, and inward near the cornea. We note that the buoyancy-driven vortex has also been observed in other computational studies in counterclockwise^30^^–^^32^ and clockwise directions.^12^^,^^33^^,^^34^ It is likely that these two counter-rotating states are both stable solutions of the problem and that either may be attained by perturbations in the boundary and initial conditions or the mesh used to solve the problem.

The AH experiences maximum flow velocities at the collector channels (1.5 × 10^−3^ m/s) due to the narrow section of these conduits (see Fig. 5b). Other than at the CCs, the AH moves fast within the buoyancy-driven vortex in the AC (4 × 10^−5^ m/s), and it is significantly slower at the TM (2.5 × 10^−6^ m/s) and CB (5 × 10^−7^ m/s). The flow velocities predicted by our model are in the same range as those of other studies.^12^^,^^34^

**Figure 6.**
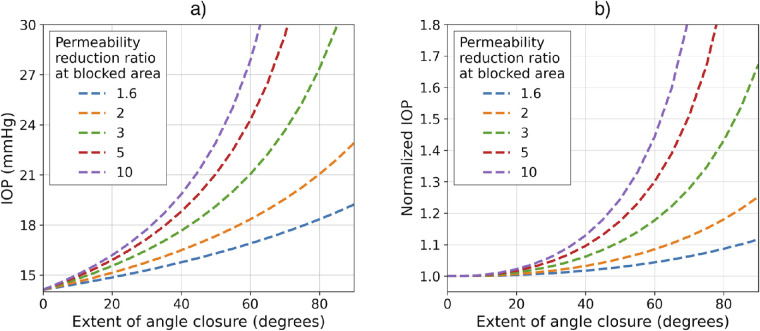
IOP in eyes with 0° of angle closure to complete angle closure. (a) IOP, and (b) IOP normalized with respect to the tangent line at the beginning of the curve. Note that an extent of angle closure of 90° in the mechanistic model represents an extent of 360° in a real eye.

Next, we modeled the relationship between the extent of angle closure and IOP (Fig. 6). As mentioned above, in our model angle closure is simulated by lowering the permeability of the regions with angle closure by a multiplicative factor (i.e., the outflow facility reduction factor). In Figure 6a, we varied this outflow facility reduction factor and the spatial extent of the closure (in degrees along the circumference) and computed the IOP value based on these conditions. As expected, we observed an increase in IOP with increasing extent of closure. This increase was nonlinear, and the degree of nonlinearity increased with increasing outflow facility reduction factor. To highlight this, we normalized this IOP to the IOP predicted by a linear increase in IOP (Fig. 6b), with a slope determined by the slope at zero angle closure in Figure 6a. If the dependence of IOP on angle closure predicted by our model was linear, the value of the normalized IOP would stay fixed at unity (normalized IOP = 1.0). However, this is not the case; instead, we noticed a deviation from linearity when the extent of closure approached 30° to 45° (30°–45° out of 90°, which represents 120°–180° out of 360° in the whole eye), depending on the outflow facility reduction factor being considered. In addition, it can be observed that the higher the outflow facility reduction factor, the higher the nonlinearity of the IOP increase.

### Clinical Data

**Figure 7.**
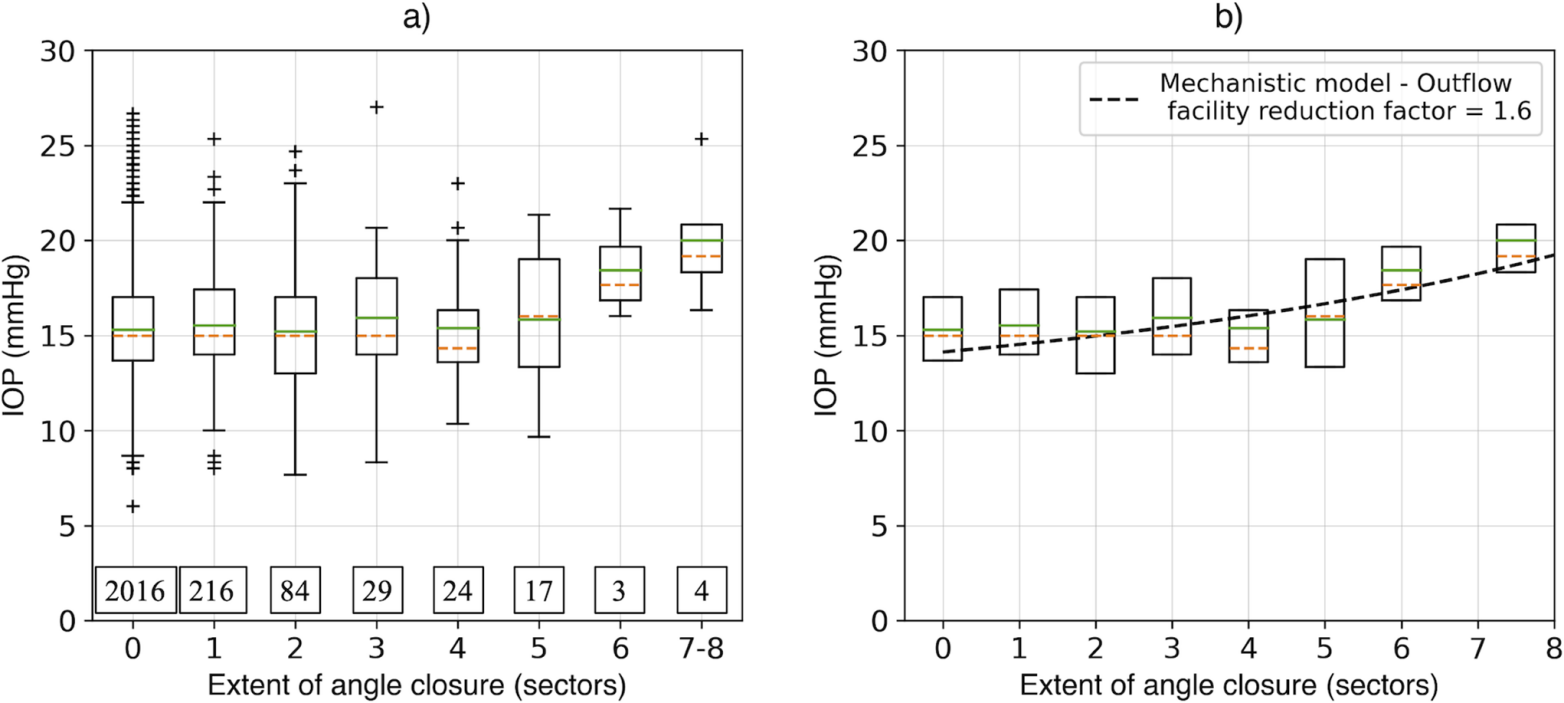
Boxplots of the IOP distribution for an increasing extent of angle closure in eyes in the clinical data. The *dashed orange line* represents the median of the distribution, the *green solid line* represents the mean, and the top and bottom of the box represent the upper and lower quartiles (Q1 and Q3). The lower whisker represents the lowest datum above (Q1 – 1.5) × (Q3 – Q1), and the upper whisker represents the highest datum below (Q3 + 1.5) × (Q3 – Q1). Boxes with no whiskers are due to limited data. The numbers in boxes represent the number of cases in each data group.

In order to examine the clinical relationship between angle closure (defined as AOD500 < 0.04 mm) and IOP, we binned each eye into nine groups, which represented the number of sectors (out of eight) where angle closure was present. Groups 7 and 8 were combined into a single group, as the number of cases in these groups was small. Figure 7a shows boxplots of the IOP distribution for the different groups, and Figure 7b compares the boxplots (without whiskers) with the mechanistic model results for an outflow facility reduction factor of 1.6. This value of the outflow facility reduction factor was selected because it best matched the clinical results. We observed that the group mean and median IOP increased with increasing number of sectors with angle closure, and that this increase was most pronounced when five or more sectors were closed.

## Discussion

In this study, we have developed a novel mechanistic model of AH flow and used it to explain the relationship between angle closure and elevated IOP. Our model includes features of the outflow pathways that are absent in previous models,^11^^,^^12^ including a model for outflow through the unconventional pathway and a decrease in TM permeability with increasing IOP. These features allow us to accurately model changes in IOP related to different physiological conditions. Specifically, we have demonstrated that our model can simulate characteristics of AH flow and IOP related to angle closure. These findings on angle closure are consistent with clinical data suggesting that IOP rise occurs when one-half of the angle is obstructed and may provide an explanation as to why only some patients with angle closure develop elevated IOP and PACG.

We used our model to examine changes in IOP caused by varying the extent of and severity of angle closure in individual eyes. The latter was modeled by reducing the permeability of the TM by a multiplicative factor (i.e., the outflow facility reduction factor) to represent scenarios in which outflow is only partially impaired due to incomplete obstruction of the TM by the iris. In all cases (Fig. 6b), we observed that the IOP rise was slow when the extent of angle closure was small (approximately less than half of the circumference) and rapid when the extent was large. This trend can be explained using a simple model for the conservation of mass, that is, *v* = *q*/*A*, where *v* is the flow velocity, *q* is the flow rate, and *A* is the “open” outflow pathway area, which represents the “open” circumferential extent of the outflow pathways. In our model, the flow rate is fixed, and an increase in the extent of angle closure leads to a linear decrease in the “open” area. However, because this term appears in the denominator of the expression for the velocity in this region, it leads to a nonlinear increase in the velocity. Finally, through Darcy's equation this leads to a nonlinear increase in the IOP, as well, which is what is observed in Figure 6. In our model, TM permeability decreases with increases in IOP, and this dependence could accentuate the increase in the IOP. However, this effect plays a relatively small role in our model compared to angle closure, as permeability is only reduced by 19% in the range of pressures considered in this study. In Dvoriashyna et al.,^35^ the effect of pupillary block (i.e., the closure of the gap between the lens and the iris) on the IOP was studied using a semi-analytical model. Remarkably, the authors observed the same nonlinear increase in IOP with pupillary block as we observed for anterior chamber angle closure, which is governed by a similar mechanistic model.

In the clinical data, we observed no apparent rise in mean/median IOP for the first five groups which represent angle closure of less than half of the circumference. However, in groups 5-8, we observe an increase in the mean IOP to approximately 20 mmHg. Qualitatively, this nonlinear increase with increasing extent of angle closure is similar to what is predicted by the mechanistic model. However, it is worth noting that the mechanistic model and clinical data examine slightly different aspects of the same phenomena. When using the mechanistic model, we examined the effect of the severity and extent of angle closure on the IOP in a *single*, simulated eye. In contrast, in the clinical data, we considered this relationship across many different eyes, and then examined how some conditional statistic of the IOP (e.g., mean) is related to the extent of angle closure. The mechanistic model reveals a nonlinear increase in IOP with the extent of closure (*e*) for all values of baseline permeability (*K*_0_) and levels of severity of closure (*S*). This implies that, if in the clinical study subjects were drawn from a distribution of the form, then

(12) $$\begin{array}{c} p\left(e,K_{0},s\right)=p_{e}\left(e\right)\bar{p}\left(K_{0},s\right), \end{array}$$

That is, if baseline permeability and the severity of closure are independent of the extent of closure, then the value of average IOP conditioned on the extent of closure will also demonstrate the same nonlinear increase observed in the computational study. From Figure 7a, it is clear that this is indeed the case.

Our findings have several important clinical and translational implications. First, IOP appears to rise rapidly when more than half of the angle is obstructed by angle closure, regardless of residual permeability to aqueous outflow. This finding supports recent clinical observations that higher IOP is observed when ITC exceeds 60% of the angle in Chinese Singaporean eyes.^36^ Knowledge about this relationship is sparse but crucial, as it could help clinicians determine when treatments that alleviate angle closure, including laser peripheral iridotomy and lens extraction, should be performed to prevent elevated IOP.^37^^,^^38^ However, precisely measuring the anterior–posterior and circumferential extent of angle closure remains challenging even with modern angle assessment methods. Gonioscopic grades are weakly correlated with IOP and AS-OCT measurements of angle width in angle closure eyes, which makes them poorly suited for measuring the extent of angle closure.^39^^,^^40^ Although AS-OCT can provide quantitative data on angle width, manual image analysis is time consuming and expertise dependent. Therefore, additional methods that support automated analysis of AS-OCT images are needed before structure–IOP relationships can be used to guide personalized treatment of angle closure eyes.^41^^,^^42^ Second, not all angle closure eyes develop elevated IOP even when angle closure is extensive. Complex factors, such as residual permeability of the iris and ciliary body to AH, may explain why the median IOP remains lower than 21 mmHg in CHES eyes with complete angle closure (Fig. 7).^43^^,^^44^ This finding serves as a reminder that the IOP cut-off of 21 mmHg to detect primary angle closure is somewhat arbitrary and that relative IOP elevations from angle closure confers higher glaucoma risk even when absolute IOP is under 21 mmHg.^45^

Our study has several limitations. First, we used an AOD500 threshold (0.04 mm) to identify angle sectors with angle closure, as ITC was not directly detected by human graders. The threshold was chosen based on the lateral resolution of the AS-OCT images. However, it is possible that eyes with angle narrowing (incomplete contact between the iris and TM) and incomplete angle closure met this definition. This may explain why models with lower permeability reduction ratios (around 1.6) are more consistent with the clinical data, suggesting that there is residual AH flow even in the presence of angle closure. We also only measured AOD500 for eight sectors of the angle, raising the possibility that other regions of the angle remained open. Second, there were only a limited number of cases of severe angle closure (seven or eight sectors) in the CHES. Although the data were sufficient to carry out a qualitative analysis of the general trend, it limited our ability to carry out statistical tests on the data. With regard to the limitations of the mechanistic model, our model assumes that the entirety of the outflow resistance of the conventional and unconventional outflow pathways lies in the TM and CB. Although this assumption is common to other studies,^11^^,^^12^^,^^32^ it is an approximation, as it circumvents the need to explicitly model complex mechanisms related to the outflow resistance such as the collapse of the SC. Also, the geometries of the TM and CB are simplified, and the permeability is considered uniform in the direction of the outflow (radial [*r*] and axial [*z*] directions). Hence, to study in detail the effects that the TM and CB have in the outflow facility, a more complex model of these tissues would be needed. Furthermore, all tissues in this model were treated as rigid. An improvement to this would be to treat them as elastic and replace the current model for the tissue with its poroelastic counterpart.^46^ Additionally, there are several improvements that can be made to make the model more realistic. These include (1) allowing for some permeability through the iris so that we have some residual flow even when the angle is fully closed, (2) using patient-specific models for the anatomy of the eye, (3) modeling outflow through the vitreous, and (4) accounting for the periodic pressure oscillations induced by the ocular pulse.

In summary, we developed a novel mechanistic model of aqueous humor outflow that improves on previous models and provides insight into the relationship between the extent of angle closure and elevated IOP. Predictions by the model are consistent with biometric and IOP data from angle closure eyes and suggest that IOP elevation occurs rapidly when more than half of trabecular meshwork is obstructed by angle closure. There are several ways in which our model could be further developed, such as modeling the TM and the unconventional outflow pathways with greater detail so that localized anatomical changes could be precisely applied to the geometric model rather than being broadly applied by changing permeability alone. In the future, this model could be more broadly applied to gain additional insights into the physiology of glaucomatous eyes, whether it is understanding mechanisms of open-angle glaucoma, in which reduced TM permeability contributes to elevated IOP, or predicting outcomes after minimally invasive glaucoma surgeries, in which outflow permeability is greatly enhanced by shunting or removal of the TM.

## Figures and Tables

**Figure A.1.**
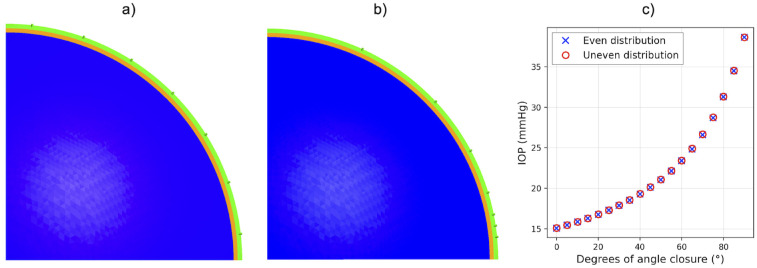
(a) Even distribution of CCS, (b) Uneven distribution of CCs, (c) Comparison of IOP levels obtained m models with even and uneven CC distributions.

**Figure B.1.**
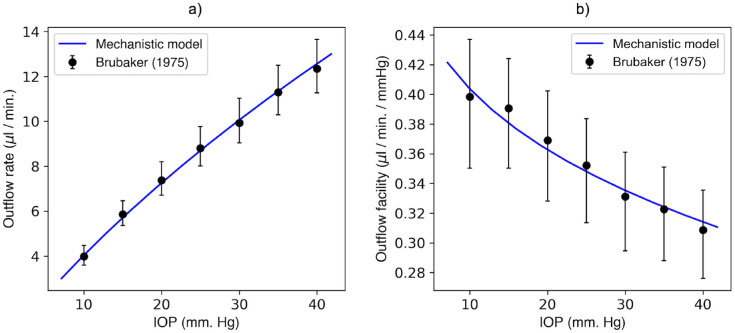
Validation of the mechanistic model results using the experimental results by Brubaker.^22^ (a) Outflow rate vs. IOP; (b) outflow facility vs. IOP.

## Appendix A Appendix A: Even Versus Uneven Distribution of Collector Channels

Various studies have previously shown segmental outflow of AH through the trabecular meshwork,^47^^,^^48^ which is believed to be caused by the morphology of the SC and CCs. It is known that CCs are randomly distributed around the circumference of the SC with higher concentrations in the inferior-nasal and temporal quadrants^49^ and that segmental flow occurs toward quadrants with higher CC concentrations.^47^ Hence, if angle closure occurs at sectors with higher outflow rates, there could be an additional increase of IOP. However, this behavior is not replicated by our model, as it considers a circumferentially continuous Schlemm's canal, which imposes near-zero flow resistance. For this reason, an uneven distribution of CCs does not affect our results. To show this, we have conducted additional simulations that verify that the distribution and location of the CCs does not affect outflow or IOP. Figure A.1a shows a geometry with evenly distributed CCs, and Figure A.1b shows the geometry with unevenly distributed CCs. In addition, Figure A.1c shows the increase in IOP for these two cases with increasing angle closure. This demonstrates that, if the SC is considered to be continuous, the distribution of CCs does not affect the IOP. Martínez Sánchez et al.^50^ arrived at the same conclusion using a similar model. Hence, for simplicity, in this study we considered an even distribution of CCs.

## Appendix B Appendix B: Model Validation

The mechanistic model was validated using the experimental results of Brubaker,^22^ which were reported for enucleated human eyes. This study was chosen because it is one of the seminal studies demonstrating the increase in outflow resistance with elevated IOP. Figure B.1 shows both the experimental results by Brubaker^22^ and the results obtained using our mechanistic model. Figure B.1a is a plot of the outflow rate as a function of IOP, and Figure B.1b is a plot of the outflow facility as a function of IOP. In both cases, we observed that the model accurately represents the trend of the experimental data; therefore, the mechanistic model can represent physiologically accurate outflow facility. The permeability parameters used in the simulations shown in Figure B.1 are listed in Table B.1.

**Table B.1.** Model Permeability Parameters

Item	Magnitude
α permeability parameter of TM, α*_TM_*	4.5 × 10^−13^ m^3^ s kg^−1^
α permeability parameter of CB, α*_CB_*	1.0 × 10^−12^ m^3^ s kg^−1^
β permeability parameter of TM, β*_TM_*	1.125 × 10^–4^ Pa^−1^
β permeability parameter of CB, β*_CB_*	3.75 × 10^–3^ Pa^−1^
